# Nanomaterial Engineered Biosensors and Stimulus–Responsive Platform for Emergency Monitoring and Intelligent Diagnosis

**DOI:** 10.3390/bios15120789

**Published:** 2025-12-01

**Authors:** Bo Fang, Yuanyuan Chen, Hui Jiang, Xiaohui Liu, Xuemei Wang

**Affiliations:** State Key Laboratory of Digital Medical Engineering, School of Biological Science and Medical Engineering, Southeast University, Nanjing 210096, China; 220242618@seu.edu.cn (B.F.); 230239535@seu.edu.cn (Y.C.); 101010998@seu.edu.cn (H.J.); 101013182@seu.edu.cn (X.L.)

**Keywords:** functional nanomaterials, biosensing, optical sensing, medical diagnosis, wearable monitoring, two–dimensional nanomaterials

## Abstract

Biosensing technology serves as a cornerstone in biomedical diagnostics, environmental monitoring, personalized medicine, and wearable devices, playing an indispensable role in precise detection and real–time monitoring. Compared with traditional sensing platforms, functional nanomaterials—by virtue of their ultra–large specific surface area, exceptional optoelectronic properties, and superior catalytic activity—significantly enhance the sensitivity, selectivity, and response speed of biosensors. This has enabled ultrasensitive, rapid, and even in situ detection of disease biomarkers, pollutants, and pathogens. This review summarizes recent advances in five key categories of functional nanomaterials—metallic, semiconductor, carbon–based, two–dimensional, and stimulus–responsive materials—for advanced biosensing applications. It elucidates the structure–property relationships governing sensing performance, such as the surface plasmon resonance of gold nanoparticles and the high carrier mobility of graphene, and analyzes the core mechanisms behind optical sensing, electrochemical sensing, and emerging multimodal sensing strategies. With a focus on medical diagnostics, wearable health monitoring, and environmental and food safety surveillance, the review highlights the application value of functional nanomaterials across diverse scenarios. Current research is progressively moving beyond single–performance optimization toward intelligent design, multifunctional integration, and real–world deployment, though challenges related to industrial application remain. Finally, the review outlines existing issues in the development of functional nanomaterial–based biosensors and offers perspectives on the integration of nanomaterials with cutting–edge technologies and the construction of novel sensing systems. This work aims to provide insights for the rational design of functional nanomaterials and the cross–disciplinary translation of biosensing technologies.

## 1. Introduction

With the continuous advancement of materials science, nanomaterials have brought revolutionary progress to advanced sensing technologies due to their unique physical and chemical properties. They significantly improve the sensitivity, selectivity, response speed, and stability of sensors, while also enabling miniaturization, flexibility, and low power consumption—thereby meeting the cutting–edge demands of various fields such as medical health, environmental monitoring, and industrial safety [1]. The intrinsic properties of different materials determine their signal transduction mechanisms as sensing media, which has greatly promoted and enriched the design of sensor devices. Table 1 summarizes the transduction mechanisms and advantageous application scenarios of mainstream nanomaterials used in current biosensing research, highlighting the classification of materials and their distinctive benefits in terms of sensing principles.

Metallic materials, such as gold nanoparticles (AuNPs) and silver nanowires (AgNWs), exhibit excellent electrical conductivity and surface plasmon resonance (SPR) effects, with resistance variation and localized surface plasmon resonance (LSPR) serving as primary sensing mechanisms [2,3,4,5,6]. Carbon–based materials, including graphene and carbon nanotubes (CNTs), possess an extremely high specific surface area, outstanding electrical properties, and mechanical flexibility, allowing them to adsorb target analytes and induce changes in the channel characteristics of resistors or field–effect transistors (FETs) [7,8,9,10,11]. Two–dimensional materials such as metal–organic frameworks (MOFs) and MXenes offer high metallic conductivity, hydrophilicity, and abundant surface functional groups. They achieve sensing through surface adsorption–induced electrical conductivity changes or synergistic effects (e.g., enhanced conductivity and oxidation resistance) when combined with other materials [12,13,14,15,16,17]. Semiconductor metal oxides and conductive polymers are particularly suitable for high–temperature gas sensing and room–temperature flexible sensing, respectively [18,19,20].

Although single–component materials exhibit unique advantages, they also present certain limitations. To overcome these drawbacks, the development of composite materials has become a major research focus [21,22]. By integrating the strengths of different material components through synergistic effects, next–generation sensing platforms with superior performance can be constructed, capable of meeting the requirements of diverse and complex application scenarios.

The limitations of conventional biosensors—typically based on macroscopic electrodes and optical labels—have become increasingly evident, including insufficient sensitivity, high limit of detection (LOD), slow response time, and limited selectivity (anti–interference capability). The incorporation of nanomaterials has provided powerful solutions to overcome these constraints, revolutionizing the design of biosensing platforms. For example, the extremely large specific surface area of carbon–based materials enhances molecular loading and diffusion efficiency, thereby improving detection limits and sensitivity [23]. Metallic materials such as gold and silver nanoparticles exhibit surface plasmon resonance (SPR) or localized surface plasmon resonance (LSPR) effects, enabling the real–time, label–free monitoring of biomolecular interactions through refractive index–dependent color shifts [24]. The surface–enhanced Raman scattering (SERS) properties of noble metals allow for the highly sensitive label–free identification of molecular “fingerprint” signals [25]. Meanwhile, the size–tunable fluorescence of quantum dots (QDs) facilitates the multiplexed detection of biomarkers [26,27]. Through these attributes, nanomaterials have driven biosensors toward higher sensitivity, lower detection limits, and enhanced selectivity.

The breakthroughs enabled by nanomaterials have addressed key technical bottlenecks that hindered the practical application of traditional biosensors, paving the way for their transition from laboratory settings to real–world use in early disease diagnosis, point–of–care testing (POCT), and in vivo imaging. Conventional diagnostic techniques—such as ELISA, PCR, and cell culture—often suffer from long processing times, operational complexity, and the need for specialized equipment and personnel. With the growing impact of chronic diseases such as cardiovascular disorders, chronic kidney disease, and cancer—which are associated with high mortality at advanced stages—early and accurate diagnosis is crucial for effective treatment and reduced patient harm [28,29]. Nanomaterial–based biosensors are designed to overcome these limitations by providing rapid, accurate diagnostic tools. Their exceptional capability for detecting disease–specific biomarkers—including glucose, sterols, nucleic acids, and proteins—highlights their significant potential in clinical applications. This review examines the unique properties of various nanomaterials, such as metallic nanostructures, carbon–based materials, MOFs, and MXenes, including size effects, surface properties, and optical, electrical, and magnetic characteristics, and highlights their practical applications in advanced sensing.

## 2. Classification and Characteristics of Nanomaterials

### 2.1. Metal Nanomaterials

Metal nanomaterials, defined as metallic substances with at least one dimension between 1 and 100 nm, represent one of the most important and extensively studied classes within nanotechnology. They are highly valued for their distinctive optical, electronic, and catalytic properties, which differ significantly from those of their bulk counterparts at the macroscopic scale. As summarized in Table 2, metal nanomaterials can be categorized into several types based on their composition and morphology. Among these, noble metal nanoparticles—such as gold and silver—have emerged as a core category due to their chemical stability and pronounced surface plasmon resonance (SPR) effects, enabling widespread applications in biosensing, imaging, and catalysis [3,30]. Metal oxide nanoparticles, including zinc oxide and iron(III) oxide, exhibit functional properties such as semiconductivity, photocatalysis, and superparamagnetism, making them suitable for uses in UV protection, environmental remediation, and medical imaging [31]. Alloy nanoparticles, which combine different metallic elements, allow synergistic performance tuning and have shown particular promise in catalysis [32]. Meanwhile, ultrasmall metal nanoclusters exhibit molecular–like discrete energy levels and strong fluorescence due to their sub–nanometer dimensions, offering new opportunities for precise fluorescence labeling [33]. Furthermore, through precise synthesis, nanostructures with controlled morphologies—such as nanorods and nanocages—enable the fine–tuning of optical properties [34,35]. For example, their plasmon resonance peaks can be shifted into the near–infrared region, making them ideal for applications such as in vivo bioimaging. By utilizing these characteristics, metal ions can provide ultra–sensitive and visualized sensing results at the microscale.

Metal–Phenolic Networks (MPNs) are a class of amorphous coordination network materials formed through the coordination between metal ions and phenolic ligands. They exhibit significant application potential in environmental emergency monitoring due to their unique physicochemical properties and environmental responsiveness. One of the most notable features of MPNs is their rapid self–assembly capability. Studies have shown that tannic acid (TA) and metal ions (e.g., Fe^3+^) can assemble on bovine serum albumin (BSA) micro–bubbles in a matter of seconds, forming stable hollow microcapsule structures [36]. This self–assembly process occurs under mild aqueous conditions, without the need for high temperature, high pressure, or organic solvents, greatly simplifying the preparation process. Compared with conventional methods, MPN fabrication avoids the hard–template dissolution step, thereby circumventing potential negative impacts on the encapsulation of functional substances caused by core dissolution.

MPNs exhibit multiple environmental response characteristics including pH and redox responsiveness [37]. These properties enable MPNs to undergo structural or functional changes in response to environmental conditions, providing a foundation for smart sensing and controlled release. In addition, MPNs demonstrate excellent biocompatibility and do not form persistent pollutants in the environment, aligning with the principles of green chemistry.

In terms of applications, the development of MPN composites and performance optimization have been key research focuses in materials science. Functionalization strategies can enhance or modify MPN properties—for instance, by incorporating magnetic properties, improving detection sensitivity, or enabling portability. Another approach involves combining MPNs with other materials to form composite systems. For example, MPN–microalgae composites not only allow for the artificial regulation of microalgal motility but also enhance the ability of microalgae to withstand environmental stress, including exposure to heavy metal ions and antibiotics [37]. In sensing platforms, the integration of MPNs with DNAzymes has enabled a novel bidirectional detection mechanism, allowing for the simultaneous detection of multiple heavy metal ions [38].

### 2.2. Semiconductor Nanomaterials

Semiconductor nanomaterials are defined as semiconductor substances in which at least one dimension is smaller than or close to their exciton Bohr radius (typically 1–20 nm). At this scale, the spatial confinement of charge carriers (electrons and holes) leads to quantized energy levels and alters the band structure, endowing these materials with physical and chemical properties distinct from those of their bulk counterparts. As a fundamental category in nanotechnology, semiconductor nanomaterials exhibit remarkable optoelectronic characteristics arising from the quantum confinement effect [39,40]. A key manifestation of this effect is the widening of the band gap as the particle size decreases, allowing the absorption and emission wavelengths (i.e., fluorescence color) to be continuously tuned without changing the chemical composition. As summarized in Table 3, semiconductor nanomaterials can be classified by dimensionality, each type offering distinct properties and application potentials. Zero–dimensional quantum dots (QDs) exhibit size–tunable fluorescence [41], superior photostability, and multiplexing capability surpassing those of conventional organic dyes. These attributes make them ideal as fluorescent probes in organelle labeling, drug delivery tracking, therapy evaluation, tumor–targeted imaging, and vascular imaging. One–dimensional nanowires (e.g., Si and ZnO), known for their efficient charge transport, are widely used in high–sensitivity field–effect transistor (FET) biosensors and nanoelectronic devices. Two–dimensional nanosheets (e.g., MoS_2_ and related transition metal dichalcogenides) possess unique band structures and are considered promising materials for optoelectronic and flexible electronic applications. Perovskite nanocrystals (e.g., CsPbBr_3_) combine high luminescence efficiency with narrow emission peaks, showing disruptive potential in displays and photovoltaics [42]. These examples illustrate that by tailoring the dimension, size, and composition of semiconductor nanomaterials, their optoelectronic properties can be precisely engineered to meet specific demands in cutting–edge fields such as biomedicine, energy conversion, and information display.

### 2.3. Carbon–Based Nanomaterials

Carbon–based nanomaterials are a class of materials composed of carbon atoms bonded via sp, sp^2^, or sp^3^ hybridization, with at least one dimension in the range of 1 to 100 nm. The versatile bonding capability of carbon allows for the formation of diverse nanostructures spanning from zero– to three–dimensional configurations, each exhibiting distinct physical and chemical properties. Thanks to their excellent mechanical strength, pronounced surface effects, superior electrical conductivity, good biocompatibility, and ease of functionalization, carbon–based nanomaterials have become ideal building blocks for customizable sensing platforms. The main types and structural characteristics of these materials are summarized in Table 4. Carbon nanotubes (CNTs), known for their high aspect ratio and exceptional electrical and mechanical properties, are widely used in composites and nanoelectronic devices [43]. Graphene, a single–layer carbon allotrope, exhibits unparalleled electrical conductivity, specific surface area, and mechanical strength, making it a cornerstone material for high–performance sensors and flexible electronics. Other carbon forms such as fullerenes, carbon dots, and nanodiamonds also offer unique advantages—including tunable fluorescence, electron–accepting capability, and outstanding biocompatibility or quantum effects—enabling their use in energy applications, bioimaging, and quantum sensing [44]. Carbon–based nanomaterials have formed structures ranging from zero to three dimensions (such as carbon nanotubes, graphene, fullerenes, etc.) through the diverse bonding modes of carbon atoms. These structures endow them with excellent electrical, mechanical, and surface properties, making them an ideal platform for constructing high–performance sensors, contributing to their broad adoption in the field of biosensing.

### 2.4. Two–Dimensional Nanomaterials

Two–dimensional nanomaterials represent one of the most prominent research frontiers in nanotechnology. They are generally defined as layered materials thinned down to a single or few atomic layers in one dimension (typically <5 nm) while extending macroscopically in the other two dimensions. In addition to the general nanoscale effects, 2D nanomaterials exhibit exceptional in–plane carrier mobility, enabling highly efficient electron transport within the plane—often surpassing the performance of conventional semiconductor materials. The electrical, optical, and catalytic properties of many 2D materials are layer–dependent [45], allowing for precise performance tuning by controlling the number of layers. The main types and structural characteristics of 2D nanomaterials are summarized in Table 5. Graphene, with its semi–metallic nature and zero bandgap, exhibits outstanding electrical and mechanical properties, making it a foundational material for transparent electrodes and high–speed electronic devices. Transition metal dichalcogenides (TMDs) compensate for graphene’s lack of a bandgap; their transition from indirect to direct bandgap in monolayer form, along with tunable electronic properties, renders them ideal for optoelectronic sensing applications [46]. The MXene family, known for its metallic conductivity and hydrophilic surface functional groups, has shown significant potential in energy storage and electromagnetic shielding. From zero bandgap graphene, bandgap adjustable transition metal disulfides to highly conductive and hydrophilic MXene, various two–dimensional nanomaterials, with their layer dependent electrical and optical properties, together forming a material system that meets the diverse application needs from electronic devices to optoelectronic sensing, energy storage, and more.

### 2.5. Stimulus–Responsive Functional Nanomaterials

Stimulus–responsive nanomaterials, often termed “smart” nanomaterials, refer to a class of nanoscale materials capable of undergoing reversible and significant physical or chemical changes in response to external stimuli such as pH, temperature, light, or magnetic fields. This stimulus–response behavior enables functions including targeted delivery, on–demand release, intelligent imaging, and feedback therapy, positioning such materials as ideal platforms for precision medicine [47]. The operational mechanism begins with the perception of a stimulus signal, leading to structural or property alterations in the material, which in turn produce a functional output. As summarized in Table 6, stimulus–responsive nanomaterials can be classified into two main categories based on the source of stimulation: endogenous stimuli (e.g., pH, enzymes, redox conditions, and temperature) and exogenous stimuli (e.g., light, magnetic fields, and ultrasound). Endogenous responsive materials can intelligently recognize disease–specific microenvironments—such as the acidic pH of tumor tissue or elevated glutathione levels—and undergo structural changes (e.g., protonation, bond cleavage) to achieve targeted drug release [48]. In contrast, exogenous responsive materials react to externally applied physical fields (e.g., near–infrared light, alternating magnetic fields) and allow for remote, spatiotemporally controlled treatments through mechanisms such as photothermal or magnetothermal effects [49]. This “sense–respond” mechanism underpins the core applications of stimulus–responsive nanomaterials in advanced fields including intelligent drug delivery, precision medicine, and theranostics. Typically, stimuli responsive materials can capture signal molecules or self–assemble with signal molecules to form nano labels, which have become powerful tools in colorimetric and fluorescent immunoassays. These smart materials can undergo controllable structural or property changes based on specific triggering factors such as pH, temperature, light, or enzymes, enabling the precise release or activation of signal reporters in situ. This not only improves the detection sensitivity and signal–to–noise ratio, but also allows for multiplexing and spatiotemporal resolution analysis, making it highly valuable for next–generation nursing point testing and clinical diagnosis.

## 3. Nanomaterial Biosensors Based on Different Detection Mechanisms

### 3.1. Optical Sensing

Optical sensing represents a core domain within biosensing technology, with broad applications spanning medical diagnosis, environmental monitoring, food safety, and beyond. When target analytes bind to biorecognition elements, they induce alterations in the local microenvironment of nanomaterials—such as refractive index, surface charge, or interparticle distance—which in turn generate measurable changes in optical signals. These changes may manifest as variations in color, fluorescence intensity, or spectral shifts, enabling the quantitative or qualitative detection of target substances.

#### 3.1.1. Local Surface Plasmon Resonance (LSPR)

**Figure 1:**
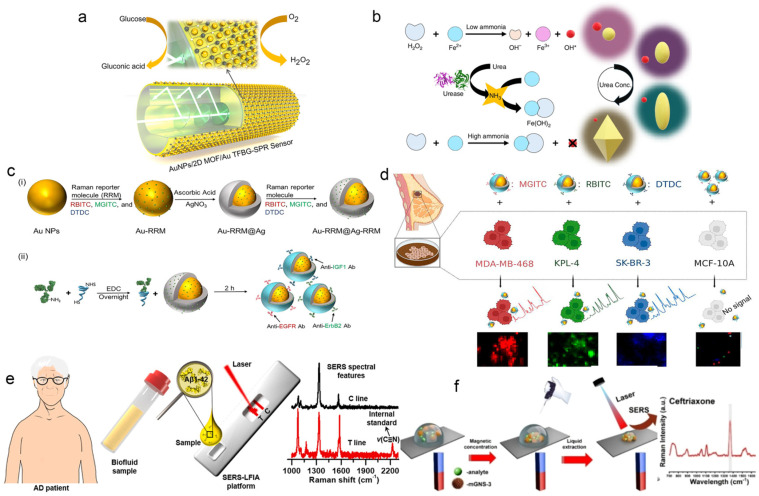
(**a**) Structure of AuNPs/2D MOF/Au TFBG–SPR sensor and glucose detection mechanism [50], the nanoenzyme constructed by MOF and AuNPs can also directly detect molecules through cascade catalytic reactions, thereby achieving dual amplification of signals and label free detection. (**b**) Schematic diagram of multi–color urea biosensor based on AuNBPs’ etching inhibition strategy [51]. (**c**) Schematic diagram of Au–Ag core–shell nanoparticles [52] (**i**) for the preparation of three different types of Au–Ag core–shell nanoparticles with Raman reporting adsorption. (**ii**) Conjugation of pegylated antibodies on the surface of the above–mentioned Au–Ag core–shell nanoparticles. (**d**) SERS imaging for the detection of phenotypic biomarkers on the cell surface membrane [52]. (**e**) Schematic diagram of the quantitative analysis of the Aβ1–42 biomarker in the biological fluid samples of AD patients by the self–calibrating SERS–LFIA biosensor. The biosensor is embedded with an internal standard (S–SERS NP) in the T–line of the test strip [53], and is used for the fluctuation correction of SERS signals. (**f**) Schematic diagram of SERS detection platform using mGNS [56].

Surface plasmon resonance (SPR) represents one of the fundamental mechanisms in optical sensing. It involves the collective oscillation of electrons at the surface of sensing material particles, with the resonance peak position being highly sensitive to changes in the refractive index of the surrounding medium. The binding of biomolecules alters the local refractive index, resulting in measurable changes such as color variation or spectral shifts (redshift/blueshift). Gold and silver nanoparticles (AuNPs/AgNPs) are the cornerstone nanomaterials for sensing applications based on this principle. For instance, studies have demonstrated that by employing two–dimensional metal–organic framework (MOF) films as substrates and enabling in situ uniform growth of AuNPs via localized surface plasmon resonance (LSPR), two dimensional MOF thin films enrich target molecules through their high specific surface area, while utilizing in situ grown gold nanoparticles (AuNPs) to generate local surface plasmon resonance (LSPR) effects to amplify optical signals, the sensor’s detection limit can be significantly lowered, allowing for ultrasensitive glucose detection without the need for additional receptors (Figure 1a) [50].

Due to this tunable optical behavior, AuNPs also play a vital role in multicolor detection and exhibit increasing functionality through surface modification. Functionalized AuNPs can undergo targeted aggregation or dispersion upon interaction with specific analytes, enabling high specificity even in complex sample matrices. A recent study by Kim et al. [51] developed a multicolor urea detection platform using modified gold nanobipyramids (AuNBPs), as illustrated in Figure 1b. The system operates via pH–controlled Fenton etching of AuNBPs to generate distinct color responses. The urease–catalyzed hydrolysis of urea releases ammonia, raising the local pH and inhibiting the Fenton reaction, thereby modulating the etching degree of AuNBPs. This approach achieves high sensitivity and a wide range of visually discernible color transitions across varying urea concentrations, with a detection limit as low as 0.098 μM—surpassing the performance of conventional colorimetric urea sensors.

#### 3.1.2. Surface–Enhanced Raman Scattering (SERS)

Surface–enhanced Raman scattering (SERS) stands as one of the most significant sensing mechanisms in optical sensing. When target molecules are adsorbed onto the surface of noble metals (such as gold or silver) or specific nanostructures, their inherently weak Raman scattering signals can be dramatically enhanced—by factors of 10^6^ to 10^14^—making even single–molecule detection feasible. The resulting Raman spectra serve as unique molecular “fingerprints”, offering exceptional sensing performance. Due to the minimal Raman signal interference from water, SERS is particularly suitable for aqueous and complex biological environments. Moreover, the distinct spectral fingerprints of different molecules enable excellent multiplex detection capability. Since its discovery, this ultra–sensitive analytical technique has remained a research hotspot in the sensing field. For example, Wang et al. demonstrated that strongly enhanced SERS signals allow for the sensitive detection of specific phenotypic biomarkers on cell surfaces [52]. Using Au@Ag core–shell nanoparticles as SERS probes, the authors embedded dual–layer Raman reporters on the gold core and silver shell, respectively. By conjugating these nanostructures with specific antibodies (Figure 1c), the highly sensitive detection of breast cancer biomarkers expressed on cell membranes was achieved (Figure 1d), along with the evaluation of chemotherapy and post–surgical outcomes. This SERS–based imaging strategy shows great potential as a powerful tool for the precise diagnosis and therapeutic monitoring of breast cancer.

In SERS sensing, most recent applications aim at detecting or quantifying one or more specific analytes through targeted analysis. This is often accomplished via an indirect detection strategy—using labeled SERS tags composed of Raman reporter molecules adsorbed onto plasmonic nanostructures. These tags bind selectively to targets via recognition elements, and the observed signal originates from the tag rather than the analyte. Lateral flow immunoassay (LFIA) represents a common platform for implementing indirect SERS detection. For instance, Li et al. developed a self–calibrating SERS–LFIA biosensor (Figure 1e) for the quantitative detection of amyloid–β (Aβ1–42) biomarkers in biofluids, enabling the accurate diagnosis of Alzheimer’s disease [53]. By embedding internal standard (IS)–encoded SERS nanoparticles into the test line as a self–calibration unit, the system corrects signal fluctuations caused by experimental variables and sample heterogeneity, significantly improving detection reliability.

The design of high–precision nanosubstrates with optimized SERS “hotspots” is crucial for enhancing signal intensity and sensing sensitivity. Localized surface plasmon resonances (LSPRs) in plasmonic nanomaterials can be excited by far–field incident light, concentrating electromagnetic energy at nanoscale features such as gaps or sharp tips [54]. These regions generate extremely strong local fields, forming the physical basis for the ultra–high sensitivity of SERS. Gold nanostars, with their multi–branched and anisotropic morphology, exhibit sharp tips (typically with curvature radii below 10 nm) capable of intense LSPR effects and electromagnetic enhancement factors reaching 10^7^–10^10^ [55]. By tuning branch length, sharpness, and density, their LSPR response can be precisely regulated across the visible to near–infrared–II (NIR–II) range.

Recently, Tuan et al. developed a direct in situ SERS detection platform using multi–branched magnetic core–shell gold nanostars (mGNS, Figure 1f) [56]. By combining the hotspot effect of gold nanostars with magnetic enrichment, this platform significantly amplifies the SERS response. Beyond intrinsic noble metal structures, constructing composite materials—such as those incorporating metal–organic frameworks (MOFs)—offers another effective route for SERS substrate design [57]. MOFs, with their high surface area and tunable porosity, can host plasmonic nanoparticles to form abundant and uniform SERS hotspots, providing additional electromagnetic enhancement [58].

#### 3.1.3. Fluorescence and Electrochemiluminescence (ECL)

Fluorescence and electrochemiluminescence represent two major branches of luminescence–based sensing technologies. Fluorescence, a form of photoluminescence, occurs when a material absorbs photons at specific wavelengths and emits lower–energy photons of longer wavelengths. Fluorescence sensing relies on changes in fluorescence signals—such as intensity, lifetime, polarization, or resonance energy transfer—resulting from interactions between target analytes and recognition elements [59]. By utilizing different fluorescent probes, this approach enables multiplex detection and offers advantages such as visual readout. Electrochemiluminescence (ECL), on the other hand, is a light–emitting process triggered by electrochemical reactions. It involves applying a voltage to an electrode to induce electron–transfer reactions between luminescent species and co–reactants in solution, generating excited–state intermediates that emit photons upon returning to the ground state [60]. As an external light source is not required, ECL directly converts electrochemical energy into optical signals, offering exceptional sensitivity, low detection limits, minimal background, high controllability, and a wide dynamic range [61]. Both fluorescence and ECL are widely applied in biomedical diagnostics, environmental safety monitoring, food testing, and life science research.

**Figure 2:**
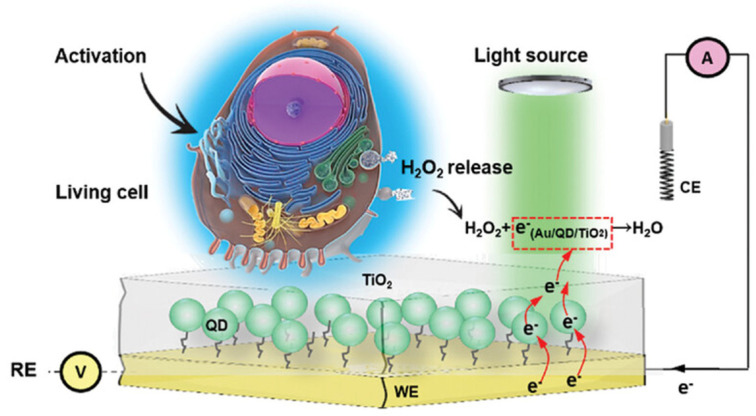
Photoelectrochemical (PEC) sensing platform for monitoring the metabolism of living cells, schematic diagram for monitoring Hela cells [62].

Quantum dots (QDs) exhibit prominent sensing mechanisms such as fluorescence resonance energy transfer (FRET) and electron transfer (ET), making them one of the most representative materials in optical sensing. As fluorescent labels, QDs offer several superior advantages over conventional organic dyes. Their broad excitation spectra allow for the simultaneous excitation of multiple QDs with a single light source, while their narrow, size–tunable emission bands enable pure multicolor coding and multiplex detection. The large Stokes shift effectively reduces background interference, and their high fluorescence brightness and exceptional photostability provide ultra–high sensitivity and strong anti–photobleaching capability, making QDs highly suitable for long–term, dynamic, and ultra–sensitive biosensing and imaging applications [61]. For instance, a study reported the development of a photoelectrochemical (PEC) sensing platform using CdSe/ZnS quantum dot (QD) electrodes for monitoring live cell metabolism [62], as illustrated in Figure 2. By constructing a QD–TiO_2_ heterojunction via atomic layer deposition (ALD), charge carrier recombination was effectively suppressed and carrier transport behavior was optimized, leading to a significant enhancement in photocurrent signal, stability, and signal–to–noise ratio. The resulting sensor exhibited micromolar–level sensitivity for hydrogen peroxide (H_2_O_2_) detection, along with excellent reproducibility, stability, rapid response, and reusability. It was successfully applied for the real–time monitoring of metabolic activity in cells grown directly on the electrode surface.

Metal–organic frameworks (MOFs) have demonstrated outstanding performance in photoluminescence (PL) sensing due to their diverse and sophisticated signal amplification mechanisms that significantly enhance detection sensitivity and selectivity. The PL signal amplification strategies of MOFs are multi–faceted and often synergistic, leveraging their structural tunability and high porosity to achieve efficient sensing. Key mechanisms include resonance energy transfer (RET) and photoinduced electron transfer (PET), which directly modulate luminescence intensity. A “Turn–On” detection mode with low background and high signal–to–noise ratio can be realized through the quencher displacement (QD) strategy. Substantial signal variations can also be triggered by structural transformation (ST) or chemical conversion (CC) processes. Furthermore, MOFs enable target enrichment and lumophore protection through channel competitive absorption (CA) or by encapsulating emitting guests such as quantum dots. Additionally, spatially confined charge transfer (CT) within MOFs facilitates luminescence enhancement and multi–color tuning [63,64]. These mechanisms often operate in concert, collectively establishing MOFs as an ideal platform for constructing highly sensitive and selective fluorescent sensors. In the design of MOF–based fluorescent sensors, careful consideration is typically given to the selection of metal centers and the design of the framework with organic ligands. For instance, certain rare–earth metals exhibit characteristic emissions; transition metals can generate fluorescence through metal–ligand charge transfer; and organic ligands with extensive conjugated systems can serve as fluorophores. To achieve superior detection performance, contemporary MOF materials often adopt composite construction strategies that integrate the advantages of different components, enabling energy transfer and synergistic sensing. As a result, MOF–based photoluminescence sensing has been widely employed in the highly sensitive detection of various targets, including biomolecular markers, pharmaceuticals, toxins, heavy metal ions, anions, explosives, and pH. In one study, a host–guest thin–film fluorescent sensor was constructed by encapsulating the fluorescent probe Me4BOPHY–1 within a ZIF–8 MOF. This approach effectively mitigated aggregation–caused quenching (ACQ) and photobleaching issues [65]. By utilizing the nanoscale cavities of the MOF to disperse the fluorophores, the fluorescence quantum efficiency was significantly enhanced from 0.76% to 19.72%. The sensor also demonstrated rapid response (3 s) and ultra–high sensitivity, with a detection limit of 1.13 ppb for the nerve agent simulant DCP.

### 3.2. Electrochemical Sensing

Electrochemical sensing, as an important branch of sensing technology, enables qualitative or quantitative analysis of target substances by measuring electrical signals—such as current, potential, or impedance—generated during electrochemical processes. In a typical measurement, the sensor (working electrode) is immersed in a solution containing the target analyte. An external instrument, such as an electrochemical workstation, applies a specific excitation signal (voltage or current) to the electrode, and the resulting response signal is recorded. The interaction between the target and the electrode surface alters this response in a manner correlated with the target concentration. Based on the type of signal measured, electrochemical sensing can be categorized into several major techniques [66,67]. Amperometric and voltammetric methods detect current changes resulting from redox reactions occurring at the working electrode, offering high sensitivity and representing the most widely applied approach. Potentiometric methods measure the potential difference between the working and reference electrodes under zero–current conditions, providing excellent selectivity and frequently being employed in ion–selective electrodes. Electrochemical impedance spectroscopy (EIS) non–destructively characterizes electrode interface properties and biomolecular interactions by analyzing the system’s impedance response to alternating current signals. Conductometric methods monitor changes in the electrical conductivity of a solution or material, featuring simple instrumentation and often being utilized in gas sensing applications.

#### 3.2.1. Label–Free Biomolecular Detection of Graphene Field–Effect Transistors (Gfet)

**Figure 3:**
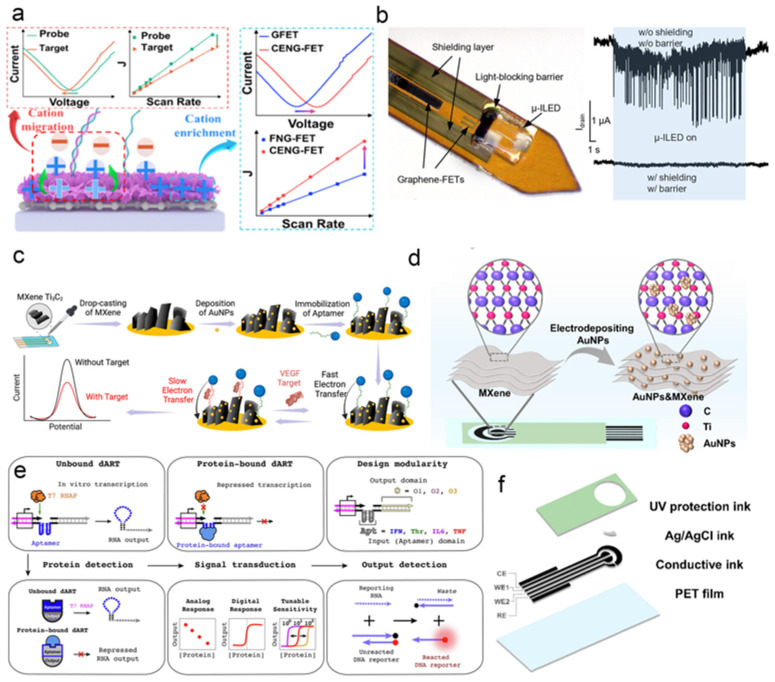
(**a**) Schematic diagram of the cationic enrichment electric field modulation strategy (CEEFMS), where the capacitance and Dirac voltage responses during the detection process are enhanced [69]. (**b**) Schematic diagram of ultra–low distortion implantable photoelectric FET bioelectronic interface [70], the combination strategy of electromagnetic shielding and light blocking barrier is adopted, effectively suppressing signal distortion caused by electromagnetic interference and photon–induced doping. (**c**) Schematic illustration of the AuNPs@MXene–empowered E–AB sensor for VEGF detection [73]. (**d**) MXene deposition was carried out to provide growth sites for AuNPs, followed by electrodeposition of AuNPs to enhance conductivity and introduce additional electroactive sites [74]. (**e**) Schematic diagram of the protein sensing platform for in vitro transcription regulated by aptamers [75]. (Above three pictures) In the absence of adapter binding, dART can transcribe and generate RNA output (**left**); protein binding inhibits this transcription process (**middle**). Its input and output domains are independent of each other, and modular design can be achieved by replacing adapters or customizing output sequences, encoding different RNA outputs (**right**). (Below three pictures) As a protein sensing layer, dARTs’ RNA output can drive downstream circuits to achieve various functions and can also generate detectable fluorescence by reacting with DNA reporter complexes. (**f**) Schematic diagrams of the structures of AuNPs and MXene–SPE [74].

Field–effect transistor (FET) biosensors represent a fundamental platform for the electrical detection of biomolecules, offering advantages such as high conductivity, label–free operation, and ease of integration. However, their development has long been limited by modest capacitance variation and the electrostatic shielding effect of counterions [68]. To address these issues, Wei et al. introduced a cationic enrichment electric field modulation strategy (CEEFMS, Figure 3a) [69] that significantly enhanced both the capacitance and Dirac voltage response during detection. The resulting CENG–FET biosensor utilizes the total capacitance variation and Dirac voltage frequency shift as output signals, demonstrating a wide linear detection range from 1 aM to 1 pM. In a separate development, Zhang et al. proposed an implantable photoelectric FET bioelectronic interface with ultra–low signal distortion [70]. As illustrated in Figure 3b, the device integrates a microscale inorganic light–emitting diode (μ–ILED) with a graphene field–effect transistor (GFET). By incorporating a light–blocking barrier and electromagnetic shielding, the system effectively minimizes interference, enabling simultaneous optical stimulation and neural recording. This work provides a foundation for the development of next–generation multimodal neural interfaces.

#### 3.2.2. Electrochemical Synergistic Effect of Gold Nanoparticle Composites

The composite of MXene and gold nanoparticles (AuNPs) has demonstrated significant potential in electrochemical sensing, largely due to their synergistic properties. MXene is a type of two–dimensional metal carbon/nitride material, serves as an ideal substrate that effectively anchors and disperses AuNPs, preventing aggregation and maintaining their small size and high catalytic activity. In return, the incorporation of AuNPs can expand the interlayer spacing of MXene, exposing additional active sites and facilitating the diffusion of reactants and products. When combined, interfacial electron transfer—typically from MXene to AuNPs—optimizes the electron density on the surface of AuNPs, significantly lowering the energy barrier of electrochemical reactions such as the reduction of H_2_O_2_ or the oxidation of target molecules [71,72]. This synergy considerably enhances the electron transfer rates and catalytic efficiency.

The in situ assembly of gold/silver nanomaterials and molecules serves as a highly effective strategy for signal amplification in electrochemical biosensors. Central to this approach is the targeted construction of metallic nanostructures directly on the electrode surface following the recognition of the analyte, thereby converting a specific biorecognition event into a substantially amplified electrical signal. This is primarily achieved through catalytic metal deposition—such as silver enhancement—where nanomaterials catalyze the reduction of silver ions to form a thick, conductive silver shell around them. The deposited silver can then be quantitatively detected via highly sensitive anodic stripping voltammetry, resulting in exponential signal amplification. This method enables detection limits as low as femtomolar to attomolar levels and has been widely applied in the ultrasensitive detection of disease biomarkers, pathogens, and environmental contaminants.

Recent studies have increasingly illustrated the practical applications of MXene–AuNP composites. For instance, a study [73] reported the first application of a gold nanoparticle–MXene Ti_3_C_2_ nanocomposite (AuNPs@MXene) in the construction of an E–AB sensor for VEGF protein detection, as illustrated in Figure 3c. The nanocomposite significantly increased the electrode’s active surface area by 30–fold and 5–fold compared to bare and AuNP–modified gold electrodes, respectively, substantially enhancing the analytical performance of the sensor during continuous monitoring. After systematic optimization, the AuNPs@MXene–based E–AB sensor demonstrated improved sensitivity and stability. In another study, an integrated portable electrochemical sensor (ip–ECS) was constructed based on AuNP–MXene–modified screen–printed electrodes (SPEs) coupled with a customized low–power electronic system for the real–time monitoring of serum biomarkers (Figure 3f) [74]. The AuNP–MXene nanocomposite significantly enhanced the electrochemical performance of the SPE by providing abundant active sites, high electrical conductivity, and superior catalytic activity (Figure 3d). The sensor was validated using model molecules such as dopamine (DA) and uric acid (UA), achieving detection limits of 1.11 μM and 1.12 μM, respectively. Furthermore, the ip–ECS was successfully applied to detect cystatin C (Cys C) in human serum, showing promising clinical utility in serum samples from pregnant women and highlighting its potential for the early risk prediction of gestational diabetes mellitus (GDM).

#### 3.2.3. Aptamer Modified Electrode Sensing

Aptamers have emerged as powerful molecular recognition elements, offering distinct advantages such as high stability, ease of modification, and a broad target spectrum. These characteristics position them as key components in the development of next–generation high–sensitivity biosensors, particularly in point–of–care testing (POCT) and on–site rapid analysis. A recent study [75] reported an innovative protein biosensing platform that operated without external instrumentation (Figure 3e). The core innovation lies in the use of aptamers to regulate transcription: by incorporating aptamer sequences into DNA transcription templates, target protein binding triggers the modulation of downstream RNA synthesis. This approach modularly transduces molecular recognition events into programmable output signals. By simply exchanging the aptamer domain, researchers can rapidly and inexpensively construct sensors for different proteins, with a tunable detection range spanning up to two orders of magnitude—potentially exceeding the intrinsic binding affinity of the aptamer itself. The system is capable of generating both analog and digital outputs, highlighting its broad potential in biotechnology and biomedical research.

### 3.3. Emerging Detection Sensing

**Figure 4:**
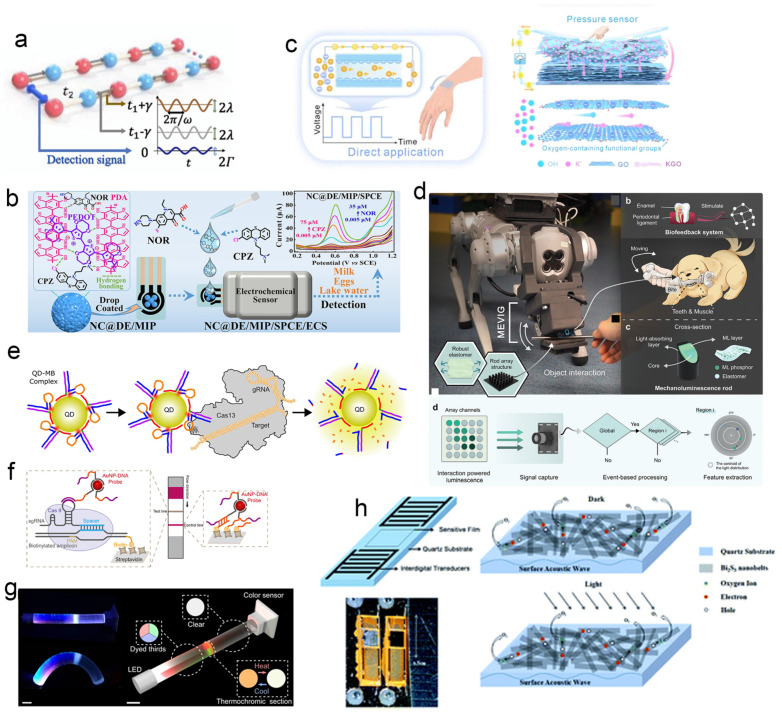
(**a**) Schematic diagram of the non–Hermitian Floquet topology sensing model (NHFTSs) [76]. (**b**) Schematic diagram of the detection principle of polydopamine–PeDot–based portable sensor [77]. (**c**) Schematic diagram of the working principle of the permeable energy direct drive flexible pressure sensor [78]. (**d**) Demonstration diagram of bionic mechanical luminescent vision touch sensor [79], inspired by the biomechanics of canine teeth. (**e**) Schematic diagram of the assembly QD MB sensing principle with hairpin complex [87]. Activated CRISPR–Cas nuclease cuts RNA hairpins. Digested hairpins release short DNA–Cy3 complements to alter the emission spectrum. (**f**) Schematic diagram of the integrated sensing system of gold nanoprobes and CRISPR–Cas for side–stream immunoassay [86]. (**g**) Schematic diagram of the sensing technology principle for omnidirectional decoding of binary changes such as bending, compression, stretching, and temperature and their combinations [88]. (**h**) Schematic diagram of high–performance visible light detector integrated with Bi_2_S_3_ nanoribbons and surface acoustic wave sensor [92].

With the continuous advancement of scientific research and industrial applications, conventional single–material architectures no longer suffice to meet the growing demands for performance and functionality. Biosensing technology is now evolving toward higher sensitivity, greater flexibility, enhanced integration, and improved intelligence. Through the adoption of novel materials and mechanisms, the detection limits of sensors are being consistently pushed forward. For instance, Zhang et al. [76] proposed a non–Hermitian Floquet topological sensing model that leverages the synergy among time–periodic driving, nonlinear dynamics, and non–Hermitian topology to achieve the ultrasensitive detection of dynamic signals (Figure 4a). This model exhibits exceptional sensitivity to dynamic boundary perturbations, with sensitivity increasing exponentially with system size. Miniaturization, integration, and portability represent major trends in the development of next–generation sensors. The TMF8829 dToF sensor from ams OSRAM, for example, enhances spatial resolution from the conventional 8 × 8 array to 48 × 32 pixels. Meanwhile, a research team from Xinjiang University developed a portable sensor based on polydopamine–PeDot composites (Figure 4b) capable of the simultaneous picomolar–level detection of chlorpromazine and norfloxacin [77]. Innovative sensing mechanisms also constitute a key focus in emerging sensor research. A team from Anhui University designed a flexible pressure sensor driven directly by osmotic energy, which generates electrical signals without an external power supply, achieving an output voltage up to 13.1 V (Figure 4c) [78]. In another study, Ding et al. [79] developed a bionic mechanoluminescent visual–tactile sensor inspired by the biomechanics of canine teeth (Figure 4d). This device emits light only under mechanical stress, enabling event–driven sensing using standard frame–based cameras, and achieved a recognition accuracy of 92.68% for interactive actions.

The integration of sensing, recognition, and computing is another significant trend in modern sensor systems. By combining sensors with AI algorithms, end–to–end intelligent perception has been realized. For example, the aforementioned osmotic energy sensor coupled with a deep learning model attained 95.78% accuracy in single–sensor gesture recognition [78]. Similarly, the bionic visual–tactile sensor achieved over 92% accuracy in recognizing eight interactive actions using a CNN algorithm [79].

#### 3.3.1. Crispr–Cas12a System Integrated with Nanomaterials

CRISPR–Cas systems, notably CRISPR–Cas9, are widely recognized for their “gene–scissor” functionality in gene editing [80,81,82]. In recent years, however, the integration of CRISPR–Cas technology with sensing platforms has emerged as a groundbreaking development in the field of biosensing, fundamentally transforming molecular detection with exceptional specificity, sensitivity, and programmability. In sensing applications, Cas proteins such as Cas12a, Cas13a, and Cas14 are often employed due to their distinct trans–cleavage activity. Upon binding to a target sequence, these enzymes become activated and exhibit promiscuous cleavage behavior, non–specifically degrading surrounding single–stranded DNA or RNA (ssDNA/ssRNA) molecules. This mechanism serves as the core signal amplification strategy in CRISPR–Cas–based sensing [83,84]. In a typical detection workflow, pre–amplified products are incubated with the CRISPR–Cas complex. If the target sequence (e.g., viral RNA) is present, Cas binding activates trans–cleavage, leading to the degradation of reporter molecules and generating a measurable signal. This approach can distinguish single–nucleotide variations—enabling applications such as pathogen strain identification and mutation detection—and, when coupled with pre–amplification, achieves detection limits as low as attomolar (aM, 10^−18^ M) levels.

Conventional CRISPR detection methods, such as SHERLOCK and DETETR, often face insufficient sensitivity, limited specificity, and usually require pre amplification steps when detecting low abundance targets, which may lead to non–specific amplification, prolonged detection time, and increased operational complexity. Conventional CRISPR–Cas biosensors often rely on fluorescently labeled ssDNA probes, which may suffer from limited stability and sensitivity in complex matrices. Although CRISPR sensing technology has shown potential in precise quantification, it still faces challenges in practical applications such as efficient in vivo delivery, immunogenicity, and off target effects. To overcome these limitations, nanomaterials including gold nanoparticles, graphene, magnetic beads, and quantum dots have been increasingly incorporated into CRISPR–Cas sensing systems to enhance performance [85]. For example, Zhou et al. [86] integrated gold nanoprobes with a CRISPR–Cas system in a lateral flow immunoassay format, successfully extending its utility to point–of–care testing (POCT, Figure 4f). In another advance, Green et al. [87] developed a quantum dot molecular beacon (QD–MB) platform that combines Förster resonance energy transfer (FRET) with CRISPR–Cas functionality, enabling sub–picomolar detection without target amplification (Figure 4e).

#### 3.3.2. Frequency–Sensitive and Multimodal Sensing

With the continuous advancement of research, single–mode sensing mechanisms—whether physical, chemical, or biological—are increasingly insufficient to meet practical application demands. “Multimodal sensing”, which integrates multiple sensing modalities into a single platform or device to simultaneously capture multidimensional and complementary information about a target, has become a major direction in next–generation sensor development. By combining different sensing principles, multimodal systems can effectively mitigate the inherent limitations of individual sensing modes. Cross–validation of multiple signals significantly reduces false positives and false negatives, thereby improving detection reliability. A core objective of advanced sensing is to detect faint changes—pursuing ultra–high sensitivity and low limits of detection capable of identifying trace amounts of target substances (even at the single–molecule level) or minute variations in physical parameters. Such capabilities are essential for detecting low–abundance biomarkers and subtle physiological signals.

For example, Paik et al. [88] reported a sensing technology that decodes multiple mechanical and thermal stimuli—including omnidirectional bending, compression, tension, and temperature changes—by analyzing the chromaticity and intensity of light transmitted through a patterned elastomer doped with functional dyes (Figure 4g). Deformation and temperature variations induce measurable changes in optical properties, enabling a one–to–one mapping between combined stimulus sequences and sensor outputs. This approach provides a basis for diverse human–computer interaction applications. In another study, Hassan et al. [89] developed a multi–frequency impedance flow cytometry system using frequency–sensitive barcoded metal–oxide Janus particles (MOJPs). By functionalizing MOJPs with antibodies targeting specific neutrophil surface markers (CD11b and CD66b), the system leverages distinct electrical impedance signatures under multi–frequency electric fields. Combined with supervised machine learning and high–speed video microscopy, it enables the precise classification of cell–particle conjugates, demonstrating powerful multi–parameter analysis at the single–cell level.

#### 3.3.3. Surface Acoustic Wave (Saw) Sensing

Surface acoustic wave (SAW) resonators are among the most prominent acoustic devices employed in chemical sensing, offering exceptional sensitivity to minute mass changes due to their ultra–high frequency characteristics [90]. By functionalizing the sensor surface with various recognition layers—including upconversion nanoparticles, metal oxide coatings, carbon nanotubes, graphene sheets, functional polymers, and biological receptors—SAW sensors have been successfully applied to detect targets ranging from small gas molecules to large biomolecules and even entire cellular structures. The advantages of SAW sensing lie in label free detection, real–time response, miniaturization potential, and ease of integration. However, its performance is affected by environmental temperature, humidity, and interface modification stability. In practical applications, material innovation and structural design are needed to overcome noise interference and selective challenges.

For instance, nanostructured palladium–platinum (PdPt) catalysts integrated with SAW devices have enabled the development of highly responsive hydrogen sensors [91]. The catalytic exothermic reaction between hydrogen and ambient oxygen generates heat, which propagates along the SAW delay path and translates hydrogen concentration into measurable electrical signals. In another study, Fu et al. [92] fabricated Bi_2_S_3_ nanoribbons via a hydrothermal method and integrated them as photosensitive materials onto an ST–cut quartz SAW delay–line sensor operating at a wavelength of 15.8 μm and a center frequency of 200.02 MHz, constructing a high–performance visible–light detector. The detection mechanism primarily arises from the mass–loading effect induced by oxygen desorption from the Bi_2_S_3_ nanoribbons under visible–light illumination (Figure 4h).

## 4. Application Fields of Biosensing Nanomaterials

### 4.1. Medical Diagnosis and Precision Medicine

Biosensing nanomaterials are extensively employed in medical detection and diagnosis, profoundly transforming healthcare by offering unprecedented capabilities in detection and therapeutic precision. Due to their unique physicochemical properties and nanoscale dimensions, these materials exhibit distinct advantages in detection specificity, the ability to cross physiological barriers, and ease of customization and integration into compact portable devices, making them a fundamental enabling technology for point–of–care testing (POCT) and in vitro diagnostics (IVD) [93].

At the diagnostic level, they function as highly sensitive and specific detection elements, widely applied in lateral flow immunochromatographic strips, liquid biopsy, medical imaging, and wearable or implantable sensors [94]. These applications enable rapid, ultra–early, and even continuous dynamic monitoring of disease biomarkers [95]. In the realm of precision medicine, nanomaterials serve as intelligent carriers, forming the basis of targeted drug delivery systems, theranostic platforms, and gene editing tools [96]. They facilitate the precise localization of pathological sites, on–demand therapy, and real–time monitoring of treatment efficacy. Collectively, these advances are driving a paradigm shift in medicine toward personalized, minimally invasive, and precision healthcare.

#### 4.1.1. Early Cancer Screening

Cancer continues to be a leading cause of mortality worldwide, driving the need for advanced diagnostic and therapeutic strategies. Nanomaterials have emerged as key enablers in oncology, particularly in early cancer screening, imaging, and targeted therapy [97]. Through surface functionalization with specific ligands, nanomaterials can achieve the highly sensitive detection of early cancer biomarkers and enable targeted drug delivery, thereby improving prevention, precise lesion localization, and reducing systemic toxicity. Due to their nanoscale dimensions and tunable surface chemistry, these materials can efficiently navigate the complex tumor microenvironment [98], allowing researchers to tailor nanomaterial–based systems according to individual patient profiles.

**Figure 5:**
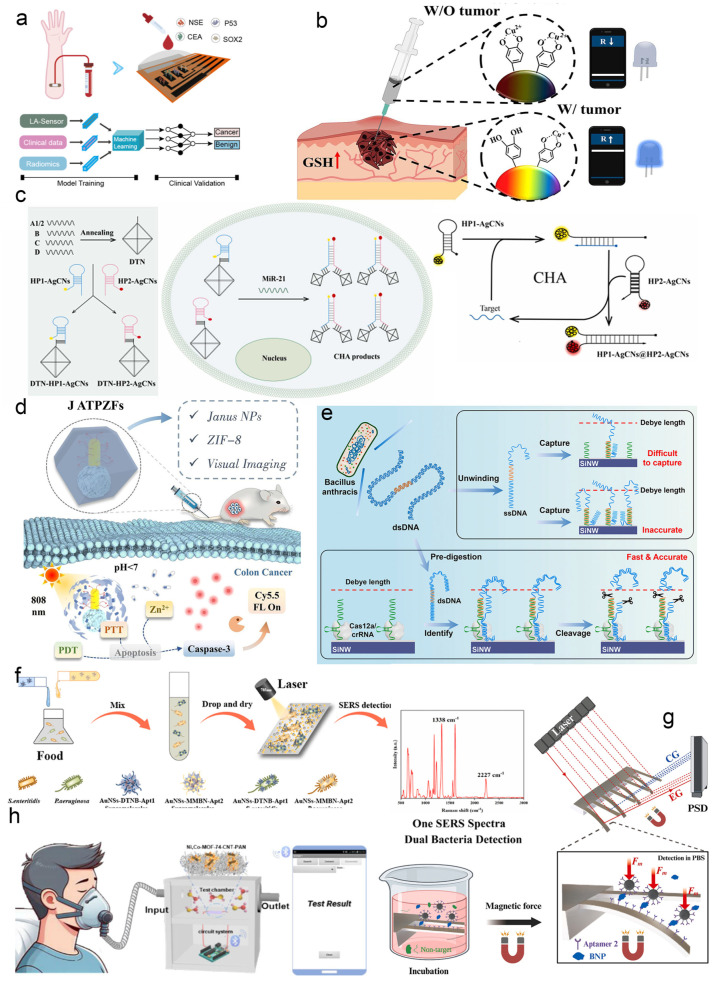
(**a**) Multiple laser–induced graphene (LIG) immunosensors combined with machine learning were used to efficiently detect four tumor markers—neuron–specific enolase (NSE), carcinoembryonic antigen (CEA), p53, and SOX2 [99]. (**b**) Detection schematic diagram of redox responsive low viscosity injectable hydrogel (LVI gel) sensor [100] integrated with glutathione (GSH) and reactive oxygen species (ROS) activated nanoparticles (PD–Cu2+). (**c**) Schematic diagram of the principle of enzyme–free nanosensors using polychromatic silver nanoclusters (AgNCs) for DNA–mediated catalytic hairpin assembly (CHA) reactions [101]. (**d**) Working schematic diagram of a novel asymmetric nanosensor for colon cancer treatment and real–time imaging [102]. (**e**) Schematic diagram of the principle of Bacillus anthracis nucleic acid detection technology [103], the Cas12a/crRNA complex precisely excises targets from long nucleic acids, mitigating issues of folding and entanglement. By fragmenting long dsDNA into segments within the Debye length, it significantly enhances sensor sensitivity, accuracy, and signal consistency. (**f**) Supramolecular biosensors based on SERS for rapid and highly sensitive detection of Salmonella enteritidis and Pseudomonas aeruginosa [104]. (**g**) Schematic diagram of the working principle of the microcantilever array in the microfluidic chip [105], detecting the nanoscale deflection of the microcantilever through the optical path. (**h**) Schematic diagram of a high selectivity and sensitivity NO sensor using Ni, Co–MOF–74–CNT–PAN sensor, detecting NO at room temperature with a LOD of 18.6 ppb [106]. It is integrated into portable devices with microcontrollers and Bluetooth for real–time, home monitoring of respiratory diseases.

In the domain of cancer detection, Wang et al. developed a multimodal early screening platform by integrating multiple laser–induced graphene (LIG) immunosensors with machine learning [99] (Figure 5a). This system combines proteomic data from immunosensors with CT imaging features and clinical information through a deep learning framework to facilitate early lung cancer screening. In a separate approach, a redox–responsive low–viscosity injectable hydrogel (LVI–gel) sensor was developed for dual–mode cancer detection [100] by integrating glutathione (GSH) and reactive oxygen species (ROS)–activated nanoparticles (PD–Cu^2+^) (Figure 5b). Upon exposure to the tumor microenvironment with high concentrations of GSH (10 mM) and ROS, structural changes in PD–Cu^2+^ led to fluorescence recovery and increased electrical conductivity, accompanied by a 95.14% reduction in hydrogel viscosity and a 360.28% enhancement in adhesion. The sensor was validated using HeLa, PC–3, and B16F10 cancer cell models, demonstrating detectable resistance changes (ΔR/R_0_ increased from 0.55 to 0.89) and wireless signal responses. This injectable platform enables ex vivo wireless monitoring of tumor biomarkers, offering a promising tool for cancer diagnosis. In the realm of cancer imaging and therapy, Ran et al. constructed an enzyme–free nanosensor based on multi–colored silver nanoclusters (AgNCs) and a DNA–mediated catalytic hairpin assembly (CHA) reaction [101] (Figure 5c), enabling high–sensitivity miRNA detection and simultaneous tumor imaging. Song et al. reported an asymmetric Janus–type nanosensor (JATPZFs) for colon cancer treatment and real–time imaging [102] (Figure 5d). The system uses a gold nanorod core partially modified with titanium dioxide and encapsulated with a folic acid–doped ZIF–8 metal–organic framework, facilitating targeted accumulation in cancer cells and responsiveness to the acidic tumor microenvironment. Under acidic conditions, the nanosensor releases zinc ions to disrupt cellular ion homeostasis and promotes reactive oxygen species (ROS) generation. It synergizes photodynamic (PDT) and photothermal (PTT) therapies to activate the caspase–3 pathway, inducing apoptosis, while allowing for the visual monitoring of treatment response via fluorescence recovery. This approach offers a promising strategy for precision oncology and therapeutic evaluation.

#### 4.1.2. Rapid Pathogen Identification

Beyond their applications in cancer diagnostics and therapy, nanosensors are increasingly employed for the rapid detection of pathogens and abnormal biomarkers. Compared to conventional laboratory methods, nano–enabled sensing offers superior sensitivity, faster response times, and greater operational convenience. In a recent study, Li et al. introduced a novel strategy for the rapid nucleic acid detection of pathogens using a crRNA–guided Cas12a system [103] (Figure 5e). This method accurately identifies and cleaves target sequences within long double–stranded DNA, effectively overcoming limitations related to nucleic acid folding and entanglement, thereby significantly enhancing detection sensitivity and accuracy. By fragmenting DNA into specific lengths uniformly distributed within the Debye length, the system ensures signal consistency. It enables attomolar (aM)–level detection of *Bacillus anthracis* DNA within 10 min. When tested on real whole–genome samples, the results showed a correlation coefficient of 0.912 compared to digital PCR, confirming the high reliability and practicality of this approach for direct pathogen nucleic acid analysis. In another advance, Tao et al. developed a supramolecular SERS biosensor [104] (Figure 5f) for the highly sensitive and simultaneous detection of two foodborne pathogens—*Salmonella enteritidis* and *Pseudomonas aeruginosa*. The sensor employs an etched triangular silicon substrate decorated with silver–gold nanoparticles (stSi@Ag–Au) as a SERS–active substrate, with further signal amplification achieved by depositing gold nanostars to create electromagnetic hot spots. Functionalization with pathogen–specific aptamers and two distinct Raman reporters (DTNB and MMBN) allows for the construction of supramolecular complexes that selectively recognize target bacteria. The assay is completed within 45 min, with a linear detection range of 10^0^ to 10^5^ CFU·mL^−1^ and limits of detection as low as 1.33 CFU·mL^−1^ for *Salmonella* and 1.12 CFU·mL^−1^ for *P. aeruginosa*.

In the domain of disease biomarker monitoring, Zhang et al. constructed a magnetic nanomechanical sensor (MNS) for the ultrasensitive detection of key cardiovascular disease (CVD) biomarkers—BNP, cTnI, and CK MB [105]. The device requires only a single drop of blood (<1 μL) and integrates a force–sensitive microcantilever with magnetic actuation to enable multi–target detection directly in blood without extensive sample pretreatment. Through surface modification to minimize nonspecific adsorption and force concentration, the sensor achieved a remarkable detection sensitivity for BNP as low as 0.1 pg·mL^−1^ (Figure 5g). Clinical validation demonstrated its ability to differentiate CVD patients from healthy individuals, highlighting its potential for long–term monitoring and the preventive management of cardiovascular and other chronic diseases. Additionally, a NO sensor based on Ni,Co–MOF–74–carbon nanotube heterostructured nanoflowers was developed for exhaled breath analysis [106]. The composite material enhances NO adsorption through its bimetallic active sites and large specific surface area. When integrated with a nanofiber membrane, the sensor exhibits high selectivity toward NO at room temperature, with a linear detection range of 30–1000 ppb and a detection limit of 18.6 ppb. Experimental results confirmed its ability to accurately distinguish exhaled NO levels between healthy individuals and patients. The sensor was successfully incorporated into a portable device equipped with a Bluetooth module, offering a promising solution for real–time at–home monitoring of airway inflammation.

#### 4.1.3. Wearable Devices

Nanomaterials are actively advancing the development of wearable flexible electronic devices and promoting the evolution of point–of–care testing (POCT) systems [107]. Conventional rigid electronic components often fail to conform adequately to the soft, dynamic surface of human skin, limiting their sensing performance. In contrast, nanomaterials—with their exceptional mechanical properties, electrical characteristics, and multifunctionality—effectively address the compatibility issues associated with rigid devices [108]. They exhibit outstanding performance in terms of device form factor, miniaturization, and wireless data transmission. For instance, graphene and carbon nanotubes offer high electrical conductivity and flexibility, making them suitable for electrophysiological monitoring and sweat–based biochemical sensing. Silver nanowires enable the fabrication of transparent and stretchable circuits, while MXene significantly enhances biochemical detection sensitivity due to its pronounced electrochemical activity. Quantum dots are well–suited for optical sensing applications, metal nanoparticles facilitate colorimetric and SERS–based detection, and nanoporous materials such as metal–organic frameworks (MOFs) excel in gas adsorption and enrichment. Together, these materials overcome the biocompatibility limitations of conventional rigid components. Leveraging their high specific surface area, superior mechanical properties, and diverse functional characteristics, they enable the development of truly flexible, comfortable, and integrated devices that maintain high sensitivity and multi–parameter detection capabilities—paving the way for breakthroughs in health monitoring, disease management, and human–computer interaction.

**Figure 6:**
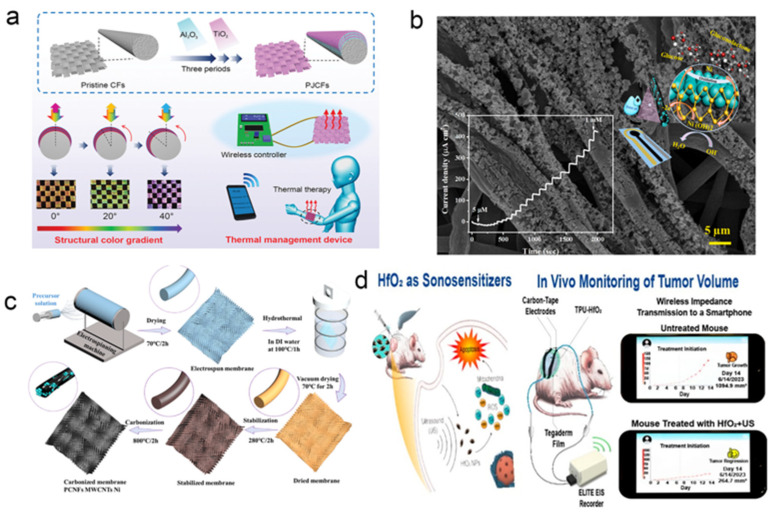
(**a**) Manufacturing process of PJCF with structural color gradient and its application in wireless wearable thermal management devices [109]. (**b**,**c**) The principle of composite materials combining 3D nanoparticle carbon–based nanofibers with magnetic nickel and MWCNT for sweat blood glucose monitoring and the schematic diagram of the composite material synthesis route [111]. (**d**) Schematic diagram of TPU–HfO_2_ [112] film as a dielectric elastomer (DE) strain sensor for real–time dynamic tracking of tumor growth and regression.

As global climate issues intensify and extreme weather events become more frequent, maintaining a stable thermal environment for the human body is increasingly critical. Excessively high or low device temperatures not only cause thermal discomfort but can also impair device performance. Effective thermal management significantly improves the wearing comfort and reliability of wearable devices. Recent progress in multifunctional nanocomposite phase change materials has accelerated such developments. For example, a novel photonic Janus carbon fiber (PJCF) has been developed [109], demonstrating a continuously gradient structural color across the visible spectrum alongside excellent mechanical durability. The material’s inherent flexibility and efficient Joule heating capability, stable under strain, allow for its integration into a wirelessly controlled system via Bluetooth (Figure 6a). This successful demonstration highlights its potential as a platform for creating next–generation, multicolor personal thermal management devices. Wearable devices also offer considerable advantages in real–time detection and long–term monitoring. Compared with traditional medical instruments, they are smaller, more user–friendly, and better suited for personalized, immediate feedback, allowing users to manage their physiological status outside clinical settings [110]. Zhu et al. developed a non–enzymatic composite sensing material for non–invasive glucose monitoring [111] (Figure 6b), fabricated via electrospinning, hydrothermal synthesis, and calcination. The material consists of three–dimensional nanoporous carbon nanofibers embedded with multi–walled carbon nanotubes and nickel particles (Figure 6c), a structure that enhances electrical conductivity, specific surface area, and electrochemical performance while promoting efficient electrocatalytic oxidation of glucose. The resulting sensor exhibits fast response, high selectivity, a wide linear range, and excellent stability, demonstrating strong potential for real–time sweat glucose monitoring.

Beyond chronic disease and physiological parameter tracking, wearable technology also shows promise in managing major conditions such as cancer. Song et al. developed an innovative wearable integrated platform for tumor monitoring and treatment [112]. The system is based on a thermoplastic polyurethane film embedded with hafnium oxide nanoparticles, which functions as a dielectric elastomer strain sensor. The film deforms proportionally with changes in tumor volume, altering its electrical impedance and enabling continuous, accurate monitoring of tumor progression and regression (Figure 6d). Simultaneously, the hafnium oxide nanoparticles act as efficient sonosensitizers, capable of killing cancer cells under ultrasound irradiation. Integrated with a wireless system, the platform transmits impedance data in real–time to a smartphone for processing and visualization, facilitating timely treatment initiation and dose optimization. By combining dynamic monitoring and therapeutic functions, this platform offers a remote, real–time, and personalized approach to cancer care.

### 4.2. Environmental and Food Safety Monitoring

**Figure 7:**
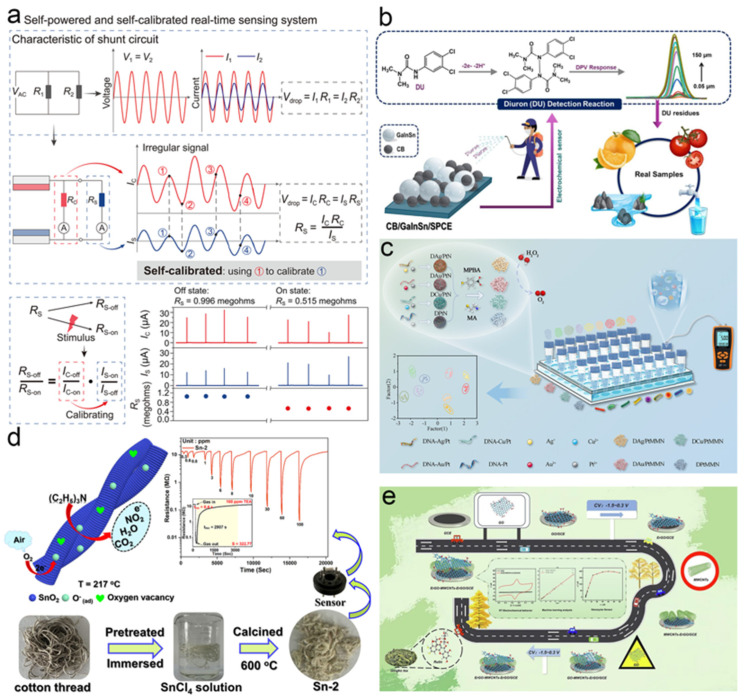
(**a**) Schematic diagram of SSEMS design and working mechanism [118]. (**b**) Schematic diagram of an electrochemical sensor for DU detection made of high–performance nanocomposites of carbon black (CB) and Ga liquid metal (GaInSn) [119]. (**c**) Schematic diagram of multiplex detection of pressure sensor arrays based on four functionalized DNA–nanoenzymes: DAg/PtN, DAu/PtN, DCu/PtN, DPtN [120]. (**d**) Schematic diagram of the sensing principle of pure–phase SnO_2_ ultrafine fibers (Sn–2) [121], pure–phase SnO_2_ ultrafine fibers (Sn–2) assembled from small–sized nanoparticles exhibit excellent triethylamine (TEA) gas sensing performance after calcination at 600 °C. (**e**) Schematic diagram of the structure and sensing mechanism of the sandwich–structured catechol nanoenzyme system (ERGO–MWcnts–ErGO) [122].

Environmental change is a prolonged and dynamic process. Tracking dynamic environmental parameters and establishing appropriate analytical frameworks are of great significance for predicting and regulating ecological systems. The distribution and concentration of environmental pollutants—such as heavy metal ions, pharmaceutical residues, pathogenic microorganisms, and toxic gases—are closely linked to environmental dynamics and serve as critical indicators for ecological forecasting and remediation [113]. Conventional detection methods often face challenges including complex sampling procedures and lengthy analysis times, making it difficult to meet the demands of modern environmental monitoring in complex and rapidly changing scenarios. As a result, nanosensing technology has been rapidly adopted in this field. The advantageous properties of nanomaterials facilitate the adsorption of target substances, signal amplification and reception, real–time data transmission, and even self–powering capabilities, thereby enabling a shift from laboratory–based analysis to in situ real–time sensing [114]. This progress supports the construction of high–density, real–time, and intelligent environmental monitoring networks. Although nanosensing applications still face several challenges, their potential remains considerable. Recent studies indicate that environmental monitoring technology is evolving toward wearable, array–based, and self–powered intelligent systems [115,116,117]. For example, Qin et al. developed a self–powered and self–calibrated environmental monitoring system (SSEMS) consisting of a triboelectric nanogenerator (TENG), calibration resistors, and a parallel sensor network for real–time temperature and humidity monitoring [118] (Figure 7a). By using calibration resistors to compensate for TENG output fluctuations caused by irregular energy inputs such as rainfall, the system achieved high–precision sensing with an error below 5.0%. In another study, the Chiu team fabricated a high–performance nanocomposite of carbon black (CB) and gallium–indium–tin liquid metal (GaInSn) via a simple ultrasound–assisted method, which was used to modify screen–printed carbon electrodes (CB/GaInSn/SPCEs) [119]. This platform served as an electrochemical sensor for detecting the persistent herbicide diuron (DU) (Figure 7b). The synergistic effect of CB and GaInSn resulted in superior electrocatalytic activity and surface–controlled electrochemical kinetics, enabling the ultrasensitive detection of DU with a limit of detection as low as 0.006 μM.

Nanosensing also plays a significant role in food safety monitoring, including the detection of foodborne pathogens, industrial quality control, and real–time tracking during food transportation. Compared to traditional methods such as chromatography–mass spectrometry, nanosensing offers greater speed, accuracy, and sensitivity. Wang et al. designed a pressure sensor array based on four functionalized DNA nanozymes to enable multiplex detection of foodborne pathogens using a portable pressure meter [120] (Figure 7c). The array exploits nonspecific interactions between functionalized DNA nanozymes (modified with 4–mercaptophenylboronic acid or β–mercaptoethylamine) and nine bacterial species, catalyzing the conversion of H_2_O_2_ to generate unique pressure–response “fingerprints”. By integrating multivariate statistical methods such as principal component analysis and hierarchical clustering, the system successfully identified and distinguished all nine pathogens with high practicality and accuracy in real samples. Xu et al. utilized waste cotton thread as a biotemplate to prepare pure–phase SnO_2_ ultrafine fibers (Sn–2) assembled from small nanoparticles through tin salt impregnation and air calcination [121]. After calcination at 600 °C, the material exhibited excellent triethylamine (TEA) gas–sensing performance (Figure 7d), attributed to its unique ultrafine fibrous morphology, mesoporous structure, high surface area, and abundant surface–adsorbed oxygen and oxygen vacancies. This sensor has been successfully applied to assess the freshness of pork, fish, and shrimp, demonstrating its potential in food quality monitoring. Additionally, a sandwich–structured catechol nanozyme system (ERGO–MWCNTs–ERGO) was constructed through the in situ self–assembly of electroreduced graphene oxide and multi–walled carbon nanotubes on an electrode surface [122], enabling the high–precision detection of rutin in Ginkgo biloba tea (Figure 7e).

### 4.3. Emerging Cross–Disciplinary Fields

Nanosensing is rapidly transcending the boundaries of traditional disciplines and achieving breakthroughs across multiple interdisciplinary fields. In intelligent bionics, it enables electronic skin to perceive touch, temperature, and humidity [123]. For brain–computer interfaces, ultra–flexible nanoelectrodes allow high–throughput and precise monitoring of neural signals [124]. In synthetic biology, nanobiosensors facilitate the dynamic tracking of metabolic processes in cellular factories [125] In space exploration, nano–enhanced spectroscopic chips have enabled in situ material analysis under extreme environmental conditions [126]. Together, these advances illustrate the evolution of nanosensing from mere “perception” toward “perception–execution–empowerment”, establishing it as a critical bridge connecting the physical, biological, and digital worlds.

**Figure 8:**
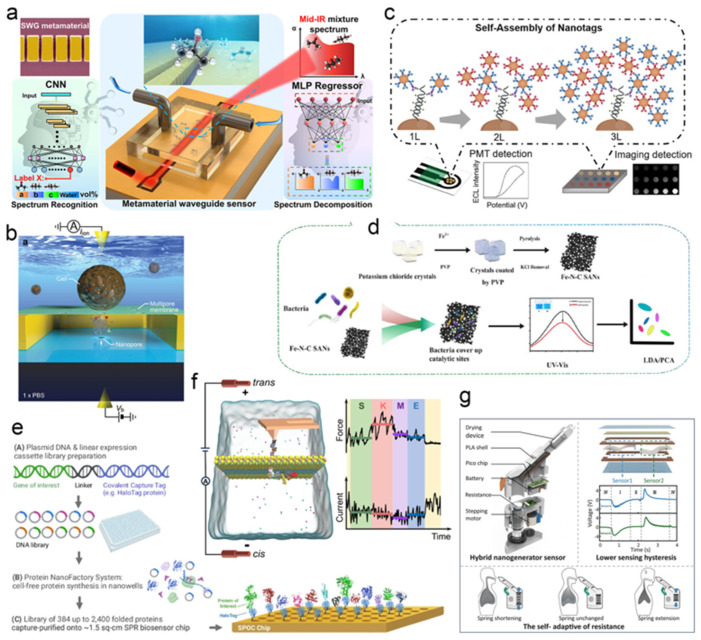
(**a**) Schematic diagram of the AI–based enhanced metamaterial waveguide sensing platform (AIMWSP) for the analysis of aqueous mixtures in MIR [127]. Using enhanced metamaterial waveguides and machine learning allows for the precise deconvolution of ternary aqueous mixture MIR spectra into individual component profiles for concentration prediction. (**b**) Schematic diagram of single–cell lysis for DNA detection by ion current measurement [130]. A voltage was applied across two vertically stacked nanopore membranes, and the corresponding ionic current was measured in phosphate–buffered saline (PBS) using a pair of Ag/AgCl electrodes. (**c**) Schematic diagram of a DNA methylation biosensing platform using DNA–functionalized magnetic beads (DNA–MBs) for target capture and self–assembly aggregation of nanotags for signal amplification [132]. (**d**) Schematic diagram of Fe–N–C SAzymes colorimetric sensor array for foodborne disease detection [128]. (**e**) Schematic diagram of the automated high–throughput platform with sensor–integrated proteomic chip (SPOC^®^) [134]. (**f**) Schematic diagram of nanoslit sensor with two–dimensional MoS2/MoSe2 planar heterostructure [133]. (**g**) Schematic diagram of an adaptive respiratory muscle trainer integrating nanosensor hybrid and artificial intelligence [129].

The integration of sensing and artificial intelligence represents a leading edge in technological convergence, bringing new momentum to intelligent recognition. Lee et al. developed an AI–enhanced metamaterial waveguide sensing platform (AIMWSP) to overcome challenges such as spectral overlap and strong water background absorption in mid–infrared (MIR) waveguide sensing for the quantitative analysis of liquid mixtures [127]. By combining highly sensitive metamaterial waveguides with machine learning algorithms, this platform successfully deconvolutes the MIR absorption spectra of ternary mixtures in aqueous media and accurately predicts the concentration of each component (Figure 8a), demonstrating great potential for on–chip MIR spectrometers in monitoring multi–component organic pollutants in water. One study developed a colorimetric sensor array that integrated Fe–N–C single–atom nanozymes with machine learning for the rapid detection of various foodborne pathogens [128]. The technique exploits the specific inhibitory effect of pathogens on the nanozyme’s activity to generate unique “colorimetric fingerprints”, enabling highly sensitive and high–throughput discrimination (Figure 8d). Demonstrating promising performance in real water samples, this approach provides a powerful tool for food safety monitoring. Recently, an adaptive respiratory muscle training system based on a triboelectric–piezoelectric hybrid nanogenerator was reported [129]. It captures high– and low–frequency breathing signals via the Kármán vortex street effect, employs a machine learning algorithm (94.4% accuracy) to monitor muscle fatigue in real–time, and uses a stepper motor to auto–adjust resistance—significantly improving the safety and efficiency of respiratory training (Figure 8g).

The intersection of nanosensing and multi–omics analysis is opening new paradigms in life science research. By delivering ultra–high sensitivity, throughput, and single–cell resolution, it enables deep mining and the integration of genomic, transcriptomic, proteomic, and metabolomic data, offering comprehensive insights into disease mechanisms. Sensors based on nanopores, nanopipettes, or nanofluidic technology allow for sampling and analysis at the single–cell level, revealing cellular heterogeneity beyond the limits of bulk sequencing. Kawai’s team developed a 3D integrated nanopore device—fabricated by vertically stacking SiO_2_ porous membranes and SiN_X_ nanopore layers on a silicon chip—for amplification–free single–cell genomic analysis [130] (Figure 8b). Applying a strong electric field (~10^6^ V·m^−1^) at the nanopore efficiently lyses individual cells, enabling the in situ detection of released DNA via ion–current measurements. The observed telegraph noise signals reflect the conformational freedom of polynucleotide chains traversing the pore, suggesting a viable chip–scale strategy for direct single–molecule sequencing and multi–omics profiling.

Multi–omics analysis is increasingly used to decipher drug resistance mechanisms. Integrating aberrant omics data helps identify resistance pathways and intervention strategies, offering critical insights for drug development and immunotherapy [131]. Nanosensors capable of detecting DNA sequences, methylation status, and therapeutic targets present new opportunities in this area. Ju et al. demonstrated a highly sensitive and universal electrochemiluminescence (ECL) platform for DNA methylation detection [132]. The assay uses DNA–functionalized magnetic beads to capture methylated DNA targets, antibody–based recognition of 5–methylcytosine (5 mC), and signal amplification via layer–by–layer assembly of antibody–conjugated [Ru(bpy)_3_]^2+^–doped silica nanoparticles, forming nanoaggregates for enhanced ECL output (Figure 8c). The platform supports both ECL measurement on screen–printed carbon electrodes and high–throughput imaging on Au/ITO microarrays, offering high sensitivity, stability, and versatility for clinical methylation analysis.

Through molecular dynamics simulations, a MoS_2_/MoSe_2_ planar heterojunction nanoslit sensor was proposed to address issues of pore clogging and uncontrolled translocation in conventional nanopore sensing [133] (Figure 8f). By confining peptide chain motion within the MoSe_2_ domain and optimizing fringe length, the device simultaneously acquires tensile force and ionic current signals, providing a theoretical basis for controllable molecular transport and high–performance peptide sequencing. Takulapalli et al. created an automated high–throughput platform named the Sensor–Integrated Proteome Chip (SPOC^®^) [134], which uses cell–free expression in nanopore arrays to synthesize and immobilize up to 2400 full–length proteins from custom DNA libraries directly on biosensor chips (Figure 8e). This system enables real–time, label–free, large–scale profiling of protein–protein interaction dynamics. With high throughput, low cost, flexibility, and minimal crosstalk, it has been applied to functional protein validation and antibody specificity assessment—including the discrimination of SARS–CoV–2 RBD variants—providing a powerful tool for drug discovery and clinical diagnostics.

## 5. Conclusions and Prospects

Nanomaterials have become indispensable in biosensing due to their unique surface effects, mechanical properties, distinctive electromagnetic characteristics, and tunable structures. Materials such as metal nanoparticles, carbon–based nanomaterials, quantum dots, nanozymes, and composites contribute to biosensing platforms with high sensitivity, operational convenience, and visual readout [135]. Through ingenious material design, a variety of sensing probes can transduce signals including fluorescence, molecular fingerprints, and electrical responses, enabling the development of optical, electromagnetic, electrochemical, and mechanical biosensors. This review has summarized the distinctive properties of major classes of nanomaterials, introduced associated sensing mechanisms, and highlighted their representative and emerging applications. Nanobiosensors are now widely employed in biomedicine, environmental monitoring, food safety, and daily life—demonstrating revolutionary performance in single–molecule detection, bioimaging, in situ tracking, self–powering, stimulus responsiveness, and highly sensitive sensing.

Current research in nanosensing is increasingly focused on updating detection mechanisms, integrating multiple functions, and enabling cross–disciplinary applications that transcend conventional technological boundaries [136]. While new nanomaterials continue to be designed at a rapid pace, scientists recognize that despite considerable progress, several challenges remain. Key technical bottlenecks still hinder the full potential of nanosensing, including limited selectivity in complex samples, interference from matrix effects, long–term stability (e.g., performance degradation beyond six months), batch–to–batch variability in synthesis, and reliability under real–world conditions—all of which pose obstacles to commercial translation.

To significantly enhance the accuracy, reproducibility, sensitivity, and practicality of nanosensors while simultaneously lowering the detection limits and costs, future research should focus on several interconnected directions. First, the innovation and optimization of nanomaterials should emphasize precise design and controllable synthesis. By regulating crystal facets, size, and morphology, batch variations can be minimized, while novel composite nanomaterials may yield synergistic effects for improved performance [137]. Additionally, multimodal sensing and data fusion strategies should be developed by integrating sensor arrays with machine learning algorithms to suppress interference and enhance detection accuracy. It is also essential to construct systems with self–calibration and adaptive capabilities to improve robustness in real–world environments [138]. Most critically, advanced computing methods such as artificial intelligence (AI) and machine learning offer powerful support for real–time data analysis, pattern recognition, and adaptive optimization of sensor operation [139]. Through these coordinated efforts, nanosensors are expected to achieve higher reliability and broader applicability, accelerating their translation into practical use.

## Figures and Tables

**Table 1:** The sensing mechanism and application scenarios of mainstream nanomaterials for biosensing.

Material Type	Representative Materials	Advantage	Sensing Mechanism
Metallic nanomaterial	AgNWs, AuNPs	Conductivity, SPR effect	Resistance variation, LSPR
Carbon–based nanomaterials	Graphene, CNTs	High specific surface area, electrical performance, mechanical flexibility	Adsorption of target substance causes changes in resistance or FET channel characteristics
Two–dimensional transition metal carbon/nitride	MXene	High metallic conductivity, hydrophilicity, and abundant surface functional groups	Surface adsorption causes changes in conductivity; Synergistic effect is generated during composite
Semiconductor metal oxide	ZnO, TiO_2_	Sensitive to gases and mature preparation processes	The target gas undergoes an oxidation–reduction reaction with the material surface, causing a change in resistance
Conductive polymer	PANI, PPy, PEDOT	Great flexibility, easy to modify	Conductivity changes during doping/dedoping process
New composite materials	Multiple combinations of materials	Synergy: Comprehensive advantages of each component	Heterojunction interface charge transfer, conductive pathway modulation, sensitization effect, inhibition of nano layer restacking

**Table 2:** The main types of metal nanomaterials.

Type	Common Materials	Properties and Uses
Noble metal nanoparticles	Au, Ag, Pt, Pd	SPR/LSPR, used for sensing and imaging; catalysis; antibacterial (Ag)
Metal oxide nanoparticles	ZnO, TiO_2_, Fe_3_O_4_	Semiconductority, photocatalysis, ultraviolet absorption, superparamagnetism
Alloy nanoparticles	Au–Ag, Pt–Ni	Tunable plasma resonance characteristics; synergistic catalytic effect, better than single metal
Metal nanoclusters	Gold clusters, silver clusters	Molecular like discrete energy levels, fluorescence emission, used for fluorescence labeling and sensing
Special morphology structure	Nanorods, nanoshells, nanostars, nanocubes, nanowires	Its plasma resonance peak strongly depends on shape

**Table 3:** The main types, common materials of semiconductor nanomaterials.

Type	Common Materials	Properties and Uses
Quantum dots (QDs)	CdSe, CdTe, PbS, InP	Size dependent fluorescence, used in biological imaging, display technology, photodetectors
Nanowires	Si, ZnO, GaN, InAs	Great electronic transmission, constructing (FET) biosensors, nanoscale electronic devices
Nanosheets	Transition metal chalcogenides (TMDs)	Unique band structure, interaction between light and matter, flexible electronic, optoelectronic devices
Perovskite nanocrystals	CsPbBr_3_, FAPbI_3_	High fluorescence quantum yield, color purity, and tunable bandgap, QLED and solar cells

**Table 4:** The main types and structural characteristics of carbon–based nanomaterials.

Type	Structural Characteristics	Properties
Graphene	Single–layer honeycomb lattice composed, sp^2^ hybridized carbon atoms arranged	High carrier mobility, high specific surface area, outstanding mechanical strength, excellent thermal conductivity, good flexibility
Carbon nanotubes (CNTs)	Single–walled carbon nanotubes (SWCNTs), multi walled carbon nanotubes (MWCNTs)	One dimensional quantum transport effect, high aspect ratio, excellent mechanical strength (>steel), high electrical and thermal conductivity
Fullerene	A football shaped hollow cage structure composed of 60 carbon atoms (C_60_)	Three dimensional electron delocalization, great electron acceptor properties, soluble in organic solvents
Carbon dots (CDs) & graphene quantum dots (GQDs)	CDs: composed of amorphous or nanocrystalline carbon nuclei and surface functional groups; GQDs: small–sized graphene sheets	Size dependent fluorescence luminescence, good photostability, high biocompatibility, easy surface functionalization
Nanodiamonds (NDs)	The diamond structure formed by sp^3^ hybridized bonding of carbon atoms	High hardness, excellent biocompatibility, chemical inertness, negative electron affinity, nitrogen vacancy color center

**Table 5:** Main types and structural characteristics of two–dimensional nanomaterials.

Type	Structural Characteristics	Properties
Graphene	Zero bandgap semimetal	High electrical and thermal conductivity, high mechanical strength
Transition metal chalcogenides	Sandwich	From indirect bandgap to direct bandgap (in monolayer), with adjustable bandgap (1–2 eV), strong light matter interaction
MXene	Two–dimensional metal carbon/nitride	High metallic conductivity, hydrophilicity, electrochemical performance
Xenes (Monoxene)	Various types of single element two–dimensional atomic crystals	Many have Dirac cones or topological properties, unstable in air

**Table 6:** Stimulus types and response mechanisms of stimulus responsive nanomaterials.

Stimulus Type	Representative Materials	Response Mechanism	Typical Applications
Endogenous stimulation			
pH	Polyelectrolytes, hydrazone bonds, ketal bonds	Materials undergo protonation, hydrolysis, or structural collapse under acidic conditions	Tumor targeted drug release
Enzyme	Peptide/protein based materials, specific enzyme substrate linking chains	The material is specifically cleaved by enzymes	Highly specific diagnosis and treatment
Redox	Polymers and selenides containing disulfide bonds (–S–S–)	Disulfide bonds and other substances break at high concentrations of GSH	Intracellular release
Temperature	PNIPAM and temperature sensitive polymers	The material has the lowest critical solution temperature (LCST)	Thermal triggered drug release
External stimulation			
Light	Gold nanorods/shells, upconversion nanoparticles, azobenzene	Specific wavelength laser irradiation produces photothermal effect or photochemical reaction	Photothermal/photodynamic therapy, remote precise controlled release
Magnetic field	Fe_3_O_4_ nanoparticles	External alternating magnetic field application causes magnetic nanoparticles to produce magnetocaloric effect	Magnetic hyperthermia, magnetic targeting
Ultrasonic wave	Microbubbles, liposomes	Ultrasonic irradiation produces mechanical or thermal effects that damage the structure of the carrier	Non–invasive trigger drug release, drug release, and gene delivery

## Data Availability

The authors do not have permission to share data.
